# Diagnostic value of chemiluminescence for urinary lipoarabinomannan antigen assay in active tuberculosis: insights from a retrospective study

**DOI:** 10.3389/fcimb.2023.1291974

**Published:** 2023-12-08

**Authors:** Luyi Huang, Yayan Niu, Li Zhang, Rong Yang, Meiying Wu

**Affiliations:** ^1^ Department of Infectious, Zhangjiagang First Peoples Hospital, Suzhou, China; ^2^ Department of Tuberculosis, The Fifth People’s Hospital of Suzhou, The Affiliated Infectious Diseases Hospital of Soochow University, Suzhou, China

**Keywords:** urine LAM antigen detection, active tuberculosis, chemiluminescence method, diagnostic value, retrospective study

## Abstract

**Purpose:**

This study aimed to assess the efficacy of chemiluminescence-based urinary lipoarabinomannan (LAM) antigen assay as a diagnostic tool for identifying active tuberculosis.

**Methods:**

A retrospective study was conducted on 166 Tuberculosis (TB), 22 Non-Tuberculous Mycobacteria (NTM), 69 Non-TB cases, and 73 healthy controls from Zhangjiagang First Peoples Hospital between July 2022 and November 2022. Clinical and laboratory data were collected, including urine samples for LAM antigen detection, sputum samples and pleural effusion for GeneXpert, TB-DNA, and culture.

**Results:**

TB group exhibited a higher LAM positivity rate (P < 0.001). CD4 count and diabetes as independent factors influencing the diagnostic accuracy of LAM. The LAM assay showed a sensitivity of 50.6% and a specificity of 95.65%. Notably, LAM’s sensitivity was superior to TB-DNA (50.60% vs. 38.16%, P < 0.05). LAM’s PTB detection rate was 51.7%, superior to TB-DNA (P = 0.047). Moreover, in EPTB cases, the LAM detection rate was 42.11%, surpassing Gene Xpert (P = 0.042), as well as exceeding the detection rates of TB-DNA and sputum culture.

**Conclusion:**

LAM antigen detection using chemiluminescence has demonstrated outstanding clinical diagnostic value for active TB, especially in the diagnosis of extrapulmonary TB. The convenience of sample collection in this diagnostic approach allows for widespread application in the clinical diagnosis of active tuberculosis, particularly in cases of EPTB and sputum-negative patients.

## Introduction

Tuberculosis (TB) remains one of the major infectious diseases that poses a serious threat to human health (Fernandes et al., 2022). According to the World Health Organization (WHO) report, the estimated number of new TB cases globally reached 10.6 million in the period from 2020 to 2021 (Bagcchi, 2023). Extrapulmonary tuberculosis (EPTB) accounts for approximately 13.37% to 53.00% of all TB cases globally, which is noteworthy (Ohene et al., 2019; Pang et al., 2019). Among the 30 countries worldwide with a high burden of TB, China currently ranks second and is also one of the countries with a high burden of drug-resistant TB (Bagcchi, 2023). Due to the coexistence of pulmonary tuberculosis (PTB) and EPTB (Ohene et al., 2019), as well as the higher drug resistance rate in EPTB compared to PTB alone (Boonsarngsuk et al., 2018), the diagnosis of these conditions is more challenging. The End TB global project proposes that the design of new diagnosis methods or the improvement of the current diagnostic tests are a priority to accelerate the efforts to stop TB (Uplekar et al., 2015).

The Common clinical tests for TB infection include Mycobacterium TB culture (Azadi et al., 2018), acid-fast smear (Azadi et al., 2018), interferon-γ release assay (IGRA) (Goletti et al., 2022), and polymerase chain reaction (PCR) (Steingart et al., 2014). Despite their advantages, these strategies have limitations, including long turnaround time, the need for sputum or invasive blood samples, reliance on specific instruments, and low sensitivity (Franzblau et al., 2012). The limitations of sputum-based TB diagnosis are particularly pronounced for individuals with EPTB or those co-infected with HIV (Franzblau et al., 2012; Zhao et al., 2012). Thus, the development of sensitive and easy-to-use urine tests for this pathogen challenge has significant implications.

The detection of lipoarabinomannan (LAM) levels in the urine represents one such possible test. LAM is a heat-stable glycolipid that is found in the outer cell wall of Mycobacterium (Brennan, 2003; Lawn et al., 2012). It is released by metabolically active mycobacteria, which makes it a valuable marker for TB detection (Minion et al., 2011). Moreover, LAM is filtered by the kidneys (Lawn et al., 2009; Minion et al., 2011), and therefore, it can be detected in urine, which overcomes the limitations of testing in patients who are unable to produce sputum. As a result, it has been recommended by the WHO (Organization, 2022). These tests have shown higher sensitivity in populations that are HIV-positive. Lawn (Lawn et al., 2012) has shown that TB-LAM testing was performed on HIV-positive patients with a CD4 cell count less than 200 cells/μL. The results showed a sensitivity of 52.5% (39.1–65.7). Another study (Székely et al., 2022) shown that Overall FujiLAM sensitivity was 54.8% (95% CI: 49.1–60.4), and overall specificity was 85.1% (83.1–86.9). However, it is disappointing that the sensitivity of this test in HIV-negative patients is low, approximately 10-20% (Achkar et al., 2011). Research on chemiluminescence platforms offering heightened sensitivity has yet to be reported.

In this study, we evaluated a novel diagnostic approach using chemiluminescence to detect the presence of LAM in the urine of HIV-negative individuals. We conducted a comprehensive analysis of the clinical factors that could influence LAM detection and performed a thorough comparison between urine LAM detection and other laboratory TB diagnostic methods, specifically focusing on the diagnosis of PTB and EPTB. The purpose of this study was to provide a new TB diagnostic method for clinical through comprehensively analyzing the clinical value of urine LAM detection and contribute to the goal of End TB.

## Materials and methods

### Study design and study population

**Figure 1:**
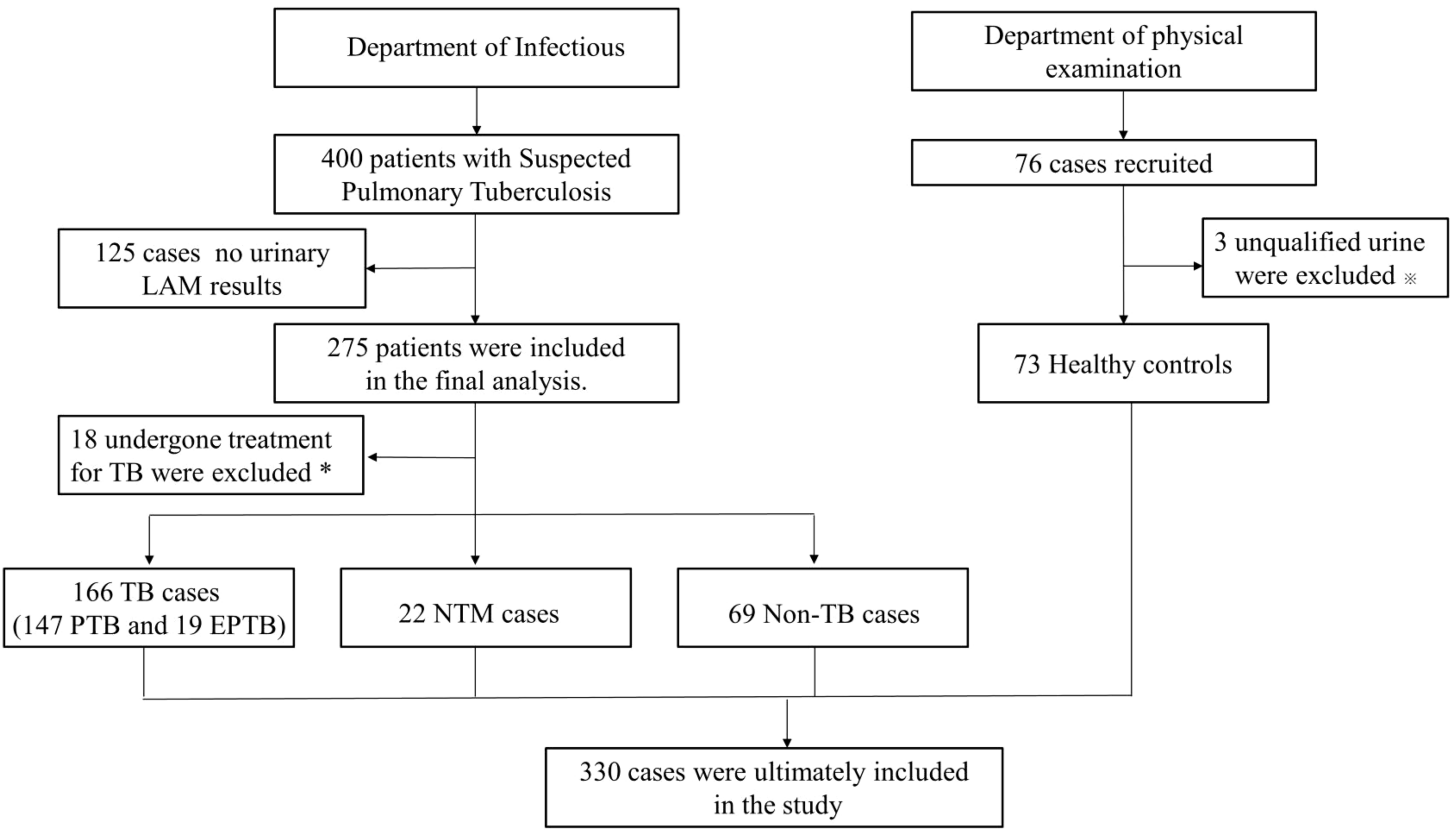
Study selection for patients flow chart. TB, tuberculosis; PTB, pulmonary tuberculosis; EPTB, extrapulmonary tuberculosis; NTM, nontuberculosis mycobacteria; Non-TB, non-tuberculosis. *Patients with previously diagnosed tuberculosis were excluded from analyses. ※Urine samples that contain suspended particles or are cloudy are at risk of contamination.

This retrospective study aimed to evaluate the diagnostic performance of urinary LAM in diagnosing TB compared to Gene Xpert, TB-DNA, and sputum culture. Between July and November 2022, a total of 400 HIV-negative suspected tuberculosis patients visited the infectious department of Zhangjiagang First People Hospital. Of these, 275 patients underwent LAM testing, and after excluding 18 individuals currently undergoing tuberculosis treatment, 257 suspected patients were included in the study. Among them, 166 were confirmed to have tuberculosis (including 147 cases of PTB and 19 cases of EPTB), 22 had NTM, and 69 were Non-TB patients. In addition, 76 individuals without TB symptoms or a history of exposure to tuberculosis were collected from the physical examination department as healthy controls and underwent LAM testing on urine samples. Three participants were excluded due to unqualified samples, leaving 73 individuals included in the healthy control group. In total, 330 participants were included in the study, all of whom were HIV-negative individuals (**Figure 1**). The diagnostic criteria outlined in the “Diagnosis for pulmonary tuberculosis (WS 288-2017)” (National Health Commission of the People’s Republic of China (2017a)) and “Classification of Tuberculosis (WS196-2017)” (National Health Commission of the People’s Republic of China (2017b)), We also referred to the “WHO consolidated guidelines on tuberculosis. Module 3: diagnosis-rapid diagnostics for tuberculosis detection” (Organization, 2020). NTM diagnostic criteria outlined in “Guidelines for Diagnosis and Treatment of Non-Tuberculous Mycobacterial Diseases (2020 Edition)” and “Consensus management recommendations for less common non-tuberculous mycobacterial pulmonary diseases” (Lange et al., 2022), The inclusion criteria for the healthy control group were individuals who underwent routine physical examinations and had no history of exposure to tuberculosis or any symptoms of tuberculosis. The study adhered to the principles outlined in the Declaration of Helsinki and received approval from the Ethics Committee of Zhangjiagang First Peoples Hospital (No. ZJGYYLL-2022-11-021). Written informed consent was obtained from all participants, allowing the utilization of their data for research purposes (**Table 1**).

**Table 1:** Baseline characteristics of participants.

Variables	TB					NTM	Non-TB	Healthy controls	χ^2^/Z	P
	LAM+	LAM-	χ^2^/Z	P	Total	n=22(6.67%)	n=69(20.91%)	n=73(22.12%)		
	n=84	n=82			n=166(50.30%)					
Gender			3.699	0.054					1.948^a^	0.378^a^
									3.246^b^	0.355^b^
Male	62(73.81%)	49(59.76%)			111(66.87%)	14 (63.64%)	52(75.36%)	45(61.64%)		
Female	22(26.19%)	33(40.24%)			55(33.13%)	8 (36.36%)	17(24.64%)	28(38.36%)		
Age (years)	56(33-67)	36.5(27-66)	-2.417	0.016	50.5(29.75-67.00)	62.82 ± 14.99	66.10 ± 17.28	57.48 ± 16.90	40.59^a^	<0.0001^a ^
Other pre-existing comorbidities										
Diabetes	17(20.24%)	5(6.10%)	5.185	0.023	22(13.25%)	2(9.09%)	13(18.84%)	_	1.78^a^	0.410^a ^
Hypertension	14(16.67%)	8(9.76%)	1.229	0.268	22(13.25%)	1(4.55%)	24(34.78%)	_	18.16^a^	<0.0001^a ^
COPD	5(5.95%)	2(2.44%)	1.268	0.26	7(4.22%)	1(4.55%)	25(36.33%)	_	46.12^a^	<0.0001^a ^
Autoimmune disease	0	1(1.22%)	1.031	0.31	1(0.6%)	0	0	_	0.55^a^	0.759^a ^
Clinical symptom								_		
Cough	69(82.14%)	60(73.17%)	1.928	0.165	129(77.71%)	19(86.36%)	66(95.65%)	_	11.43^a^	0.003^a ^
Hemoptysis	8(9.52%)	12(14.63%)	1.108	0.292	20(12.05%)	0	7(10.14%)	_	3.01^a^	0.222^a ^
Hyperthermia	9(10.71%)	9(10.98%)	0.095	0.758	18(10.84%)	1(4.55%)	17(24.64%)	_	9.49^a^	0.009^a ^
Methods										
LAM	84(100%)	82(100%)	0.024	0.877	166(100%)	22(100%)	69(100%)	73(100%)		
Gene Xpert	78(92.86%)	78(95.12%)	0.376	0.54	156(93.98%)	22(100%)	67(97.10%)	_		
TB-DNA	75(89.29%)	77(93.90%)	1.145	0.285	152(91.57%)	22(100%)	67(97.10%)	_		
Sputum culture	75(89.29%)	77(93.90%)	1.145	0.285	152(91.57%)	18(81.82%)	68(98.55%)	_		
CD4 count (*10^9^ cells/L)	0.46 ± 0.24	0.52 ± 0.24	0.616	0.003	0.51 ± 0.25	0.46 ± 0.19	0.57 ± 0.26	_	2.04^a^	0.361^a^
CD8 count (*10^9^ cells/L)	0.38 ± 0.22	0.41 ± 0.22	0.621	0.05	0.41 ± 0.22	0.38 ± 0.17	0.46 ± 0.21	_	1.67^a^	0.434^a^
CD4/CD8	1.43 ± 0.70	1.61 ± 1.51	1.397	0.317	1.52 ± 1.17	1.36 ± 0.64	1.38 ± 0.83	_	0.64^a^	0.726^a^

TB, tuberculosis; NTM, nontuberculosis mycobacteria; Non-TB, non-tuberculosis; LAM, Liboarabinomannan; CPOD, Chronic Obstructive Pulmonary Disease; TB-DNA, Mycobacterium tuberculosis gene amplification assay; _, not collected; ^a^, compared among TB, NTM and Non-TB groups; ^b^, compared among TB, NTM, Non-TB and Healthy controls groups; P<0.05, significant difference.

### Sample collection

#### Sputum samples

Sputum samples were collected from the patients through spontaneous expectoration. Fresh sputum was obtained from the patients’ first-morning expectoration after rinsing their mouths with water. The patients were instructed to forcefully expectorate from deep within their lungs into a glass or plastic cup or onto wax-coated paper. In cases where patients had no or minimal sputum, they underwent inhalation of a mist of saline solution (100 g/L) heated to 45°C to aid in the expectoration of sputum.

Three sputum samples were collected from each patient, including one in the evening, one in the early morning, and one at the time of collection. These three samples were combined and thoroughly mixed to form a single specimen. Subsequently, the sputum specimen was mixed with 5 mL of 0.9% saline solution to ensure uniformity and facilitate laboratory processing. The prepared specimen was then sent to the laboratory for further analysis.

#### Urine samples

When collecting urine samples from patients, we should adhere to the following conditions: Each test sample should have a total urine volume of no less than 10 mL. When selecting midstream urine samples, it is important to avoid samples with high protein content, fat, or other interfering substances in order to ensure sample accuracy. After sample collection, the specimens should be stored at room temperature, and the storage time should not exceed 72 hours. If testing cannot be completed promptly, the samples should be refrigerated at 2°C.~8°C for a maximum of 7 days. If long-term testing is delayed, the samples should be stored at -15°C or below. Additionally, to prevent damage to the sample, it is recommended to minimize the number of freeze-thaw cycles, and the number of freeze-thaw cycles should not exceed three.

### Pleural effusion

Before performing thoracentesis, healthcare professionals communicate with the patient, explaining the purpose, steps, and potential risks of the procedure, and obtain the patient’s consent. Clinicians typically use clinical examinations and imaging studies, such as chest X-rays or ultrasounds, to determine the location and nature of the pleural effusion. Local anesthesia and disinfection are applied at the sampling site to alleviate the patient’s pain and minimize the risk of infection. A fine needle or catheter is used by the doctor to puncture the site of the pleural effusion, and the pleural fluid is aspirated into a collection container through the needle or catheter. Once the fluid is collected, the needle or catheter is carefully removed, and the sampling site is treated appropriately.

### Laboratory TB diagnostic methods

#### GeneXpert

According to the instructions, the processing solution was mixed with the sputum sample or pleural effusion at a ratio of 1:2. The mixture was then placed on a vortex mixer and vortexed for 0.5 minutes. After incubating at a specific temperature for 15 minutes, the specimen was vortexed again for 0.5 minutes and then incubated for an additional 5 minutes until it was fully liquefied. The mixed solution was then added to the detection cartridge and placed in the GeneXpert instrument for testing. The GeneXpert detection system and accompanying reagents were provided by Cepheid, USA.

#### TB-DNA-PCR

The drainage of Sputum or pleural effusion were placed in a sterile sputum box for examination. The TB-DNA detection kit from Sun Yat-sen University Da’an gene Co., Ltd. was used, and the detection process was carried out following the provided instructions.

#### Mycobacterium tuberculosis culture

Sputum specimens or pleural effusion underwent a decontamination process using N-acetyl-L-cysteine-sodium hydroxide (NALC-NaOH) solution. Subsequently, the specimens were washed with a phosphate buffer at pH 6.8. A volume of 0.5 mL from each specimen was then inoculated into MGIT (Mycobacteria Growth Indicator Tube) culture tubes, which contained nutrient supplements and antimicrobial agents. To facilitate culture and detection, the culture tubes were placed in a BACTEC MGIT 960 Mycobacterial Detection System. The process of mycobacterial isolation and culture examination followed the guidelines outlined in the “Laboratory Testing Guidelines for Tuberculosis Diagnosis.” The MGIT 960 detection system and its associated reagents were provided by BD, USA.

#### Urinary LAM detection (chemiluminescence)

For urinary LAM detection, 4 mL of midstream urine was collected from the patient, and the test was performed following the instructions provided by the manufacturer (Guangzhou Leide Biotechnology Co., LTD., China). 1.5 mL of the test sample was transferred to a 10 mL centrifuge tube. Then, 50 µL of the magnetic bead reagent, which contains LAM-capturing antibodies, was added to the centrifuge tube and mixed. The tube was labeled for identification. The labeled centrifuge tube was placed in a rotating mixer and incubated at room temperature with a rotation speed of 30-50 rpm for 2 hours. After incubation, the centrifuge tube was placed on a magnetic rack for adsorption. Once the components were fully separated, the liquid above the sediment was discarded. The mixture was thoroughly mixed using a vortex mixer. Within 5 minutes, the sample was processed following the operation manual of the LAM detection chemiluminescence analyzer. If the time exceeds 5 minutes, the sample needs to be mixed again before testing. The LAM detection system and accompanying reagents were provided by Leide Biosciences Co., Ltd, China.

### Statistical analysis

Statistical analysis was conducted using SPSS 20.0. Baseline characteristics are presented as means ± standard deviation (SD) or as medians [interquartile ranges (IQRs)] for continuous variables, as appropriate. Categorical variables are presented as numbers (percentages). For categorical variables in the clinical and demographic data, the chi-squared test, Student’s t-test, and Mann-Whitney U test were employed to compare differences between categorical variables, normally distributed continuous variables, and non-normally distributed continuous variables, respectively. Logistic regression analysis was utilized to evaluate the relationship between measured variables and the diagnosis of LAM.

## Results

### Patient characteristics

In our study, we analyzed a total of 330 cases. The demographics and clinical characteristics of the final study population were presented in **Table 1**. Among these cases, 166 (50.30%) were diagnosed with TB, 22 patients (6.67%) had NTM infections, 69 (20.91%) had Non-TB conditions (pulmonary diseases other than TB), and 73 patients (22.12%) formed the healthy control group without pulmonary diseases (**Table 1**). The gender distribution was similar across the TB, NTM, Non-TB, and healthy control groups (P=0.355), as well as within the TB, NTM, and Non-TB groups. The TB group had a significantly younger age compared to the other groups (P<0.0001). We also examined the prevalence of other existing conditions among the TB, NTM, and Non-TB groups, and found that the rates of diabetes (P=0.410) and autoimmune disease (P=0.759) were similar. However, hypertension (P<0.0001) and COPD (P<0.0001) were significantly more common in the Non-TB group than in the other groups. Analyzing clinical symptoms, we noted that the occurrence of cough (P=0.003) and hyperthermia (P=0.009) was notably higher in the Non-TB group compared to the TB and NTM groups. CD4 count(P=0.361), CD8 count (P=0.434), and CD4/CD8 ratio (P=0.726) showed no significant differences among the TB, NTM, and Non-TB groups.

In the TB group, 84 cases were LAM+ (50.6%), while 82 were LAM- (49.4%). The LAM+ group had a higher average age (56 (33–67) vs 36.5 (27–66), P=0.016) and a greater prevalence of diabetes (20.24% vs 6.10%, P=0.023) compared to the LAM- group. However, the CD4 count in the LAM+ group was significantly lower than in the LAM- group (0.46 ± 0.24 vs 0.52 ± 0.24, P=0.003).

### Diagnostic performance of LAM

**Figure 2:**
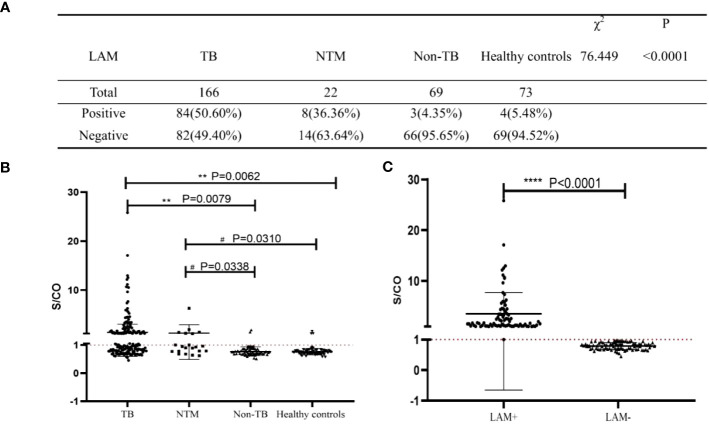
Analysis of the diagnostic performance of LAM. **(A)** The diagnostic results of LAM in the four study groups. **(B, C)** Statistical analysis of S/CO. S/CO, S represents the optical density of sample, with CO referring to the Cut Off, which is the critical value. If the S/CO value is greater than 1, it indicates that the specimen produced a result higher than the critical value, and therefore it is considered positive. Conversely, if the S/CO value is less than 1, it indicates that the specimen produced a result lower than the critical value, and therefore it is considered negative. **, P<0.01; ****, P<0.0001, compared to TB Group; #, P<0.05, compared to NTM Group; significant difference.

Based on the reference range provided by the test kit (S/CO>1 is positive), we compared the LAM positive and negative rate among these groups. Among TB, NTM, Non-TB and healthy control group, the positive rate of LAM in TB group significantly higher than other groups (50.6% vs. 36.36% vs. 4.35% vs. 5.48%, P<0.0001, **Figure 2A**). Next, we compared the S/CO level among these groups, and found that the S/CO level in TB group was significantly higher than that in non-TB group (2.22 ± 3.33 vs 0.78 ± 0.19, P=0.0079, **Figure 2B**) and healthy group (2.22 ± 3.33 vs 0.76 ± 0.14, P=0.0062, **Figure 2B**), the S/CO level in NTM group was significantly higher than that in non-TB group (2.78 ± 7.43 vs 0.78 ± 0.19, P=0.0338, **Figure 2B**) and healthy group (2.78 ± 7.43 vs 0.76 ± 0.14,P=0.0310, **Figure 2B**), but have no difference between TB group and NTM group (2.22 ± 3.33 vs 2.78 ± 7.43,P=0.7977, **Figure 2B**). We divided the TB group into LAM- and LAM+ groups based on the reference range and found that the S/CO levels of LAM+ were significantly higher than those of LAM-(**Figure 2C**).

### Analysis of risk factors of LAM

To clarify the risk factors that influence the diagnostic results of LAM, we conducted a univariate logistic regression analysis on the significant variables that showed differences between the LAM- and LAM+ subgroups in TB patients (**Table 2**). The univariate analysis revealed that age (Odds Ratio [OR]: 1.021, 95% Confidence Interval [CI]: 1.01-1.04, P=0.011), diabetes (OR: 3.91, 95%CI:1.37-11.16, P=0.011), and CD4 count (OR: 0.13, 95%CI:0.03-0.54, P=0.005) were significantly associated with the LAM diagnostic results. But CD8 counts have no correlation with LAM diagnostic (OR: 0.24, 95%CI:0.05-1.02, P=0.054). After for confounders (age, diabetes and CD4 count), patients with diabetes exhibited a three-fold probability of LAM-positive (OR: 3.14, 95%CI:1.06-9.29, P=0.039), and patient with increase in CD4 count, the probability of being LAM-positive decrease (OR: 0.197, 95%CI:0.046-0.85, P=0.029).

**Table 2:** Univariate and multivariable analysis of the diagnosis of LAM in TB.

Variables	Univariate analysis			Multivariate analysis		
	β	OR [95% CI]	P	β	OR [95% CI]	P
Age	0.02	1.021(1.01-1.04)	0.011	0.014	1.015(1.00-1.03)	0.096
Diabetes (yes vs no)	-1.117	3.91(1.37-11.16)	0.011	1.144	3.14(1.06-9.29)	0.039
CD4 count	-2.02	0.13(0.03-0.54)	0.005	-1.624	0.197(0.046-0.85)	0.029
CD8 count	-1.44	0.24(0.05-1.02)	0.054			

Variables with P≤ 0.05 in univariate models were analyzed in the multivariate analysis model. OR, Odds ratio; CI, Confidence interval.

### Comparison the diagnostic performance of 4 methods in TB

To assess the diagnostic accuracy of LAM, we compared LAM with Gene Xpert, TB-DNA, and sputum culture. According to the composite reference standard (CRS), the sensitivity, specificity, positive predictive value (PPV), negative predictive value (NPV), and Kappa value of Gene Xpert for detecting active pulmonary tuberculosis were 55.13%, 100%, 100%, 48.91%, and 0.425, respectively. The corresponding values of TB-DNA were 38.16%, 100%,100%, 41.61%, and 0.274, respectively. The corresponding values of Sputum culture were 46.71%, 100%, 100%, 45.64%, and 0.351, respectively. The corresponding values of LAM detection were 50.6%, 95.65%, 95.55%, 44.59%, and 0.347, respectively. The sensitivity of LAM was significantly higher than TB-DNA (50.60% vs 38.16%, P<0.05), slightly higher than Sputum culture (50.60% vs 46.71%) and slightly lower than GeneXpert (50.60% vs 55.13%) (**Table 3**). We also investigated the diagnostic accuracy of four methods for NTM and found that sputum culture had the highest sensitivity for NTM diagnosis (72.22%), followed by LAM (36.36%), as shown in **Supplementary Table 1.**

**Table 3:** The diagnostic performance of four methods in TB.

Methods	Results	CRS		Sensitivity	Specificity	PPV	NPV	Kappa
		TB	Non-TB					
LAM	positive	84	3	50.60%	95.65%	95.55%	44.59%	0.347
	negative	82	66					
Gene Xpert	positive	86	0	55.13%	100%	100%	48.91%	0.425
	negative	70	67					
TB-DNA	positive	58	0	38.16%*	100%	100%	41.61%	0.274
	negative	94	67					
Sputum culture	positive	71	0	46.71%	100%	100%	45.64%	0.351
	negative	81	68					

CRS, composite reference standard; TB, tuberculosis; Non-TB, non-tuberculosis; LAM, Liboarabinomannan; TB-DNA, Mycobacterium tuberculosis gene amplification assay; PPV, positive predictive value; NPV, negative predictive value. Sensitivity = True Positive Cases/(True Positive Cases + False Negative Cases) × 100%; Specificity = True Negative Cases/(True Negative Cases + False Positive Cases) × 100%; PPV = True Positive Cases/(True Positive Cases + False Positive Cases) × 100%; NPV = True Negative Cases/(True Negative Cases + False Negative Cases) × 100%; *, P<0.05, compared to LAM detection.

### Comparison the detection rates of 4 methods in PTB and EPTB

Simultaneously, we compared the detection rates of these four diagnostic methods in PTB and EPTB (**Table 4**). In PTB, the detection rate of LAM was significantly higher than that of TB-DNA (51.7% vs 40%, P=0.047), and have no significant difference with Gene Xpert (51.7% vs 60.43%, P=0.137) and sputum culture (51.7% vs 48.57%, P=0.596). In EPTB, the detection rate of LAM was significantly higher than that of Gene Xpert (42.11% vs 11.76%, P=0.047), and slightly higher than that of TB-DNA (42.11% vs 16.67%, P=0.129) and sputum culture (42.11% vs 25%, P=0.326). However, due to the relatively small sample size, no significant differences were observed.

**Table 4:** The detection rate of four methods in PTB and EPTB.

Methods	PTB			EPTB		
	n	Detection rate	P	n	Detection rate	P
LAM	147	76 (51.70%)	-	19	8 (42.11%)	-
Gene Xpert	139	84 (60.43%)	0.137	17	2 (11.76%)	0.042*
TB-DNA	140	56 (40.00%)	0.047*	12	2 (16.67%)	0.129
Sputum culture	140	68 (48.57%)	0.596	12	3 (25.00%)	0.326

PTB, pulmonary tuberculosis; EPTB, extrapulmonary tuberculosis; LAM, Liboarabinomannan;TB-DNA, Mycobacterium tuberculosis gene amplification assay; *, P<0.05, compared to LAM assay.

## Discussion

Currently, it is necessary to perform several tests to have a TB diagnosis. Despite the availability of new diagnostic techniques, the smear test and the culture of the microorganism are the gold-standard tests, but these are delivering late diagnoses (Broger et al., 2023). The LAM urine assay was seen as a potentially revolutionary diagnostic for active TB (Flores et al., 2021). With its potential to be used as a simple point-of-care test, lack of bio-safety concerns, and use of a noninvasive, convenient patient specimen, the LAM assay was fast-tracked for commercial development (Flores et al., 2021). The existing commercial LAM detection kits, due to the limitations of detection methodology (colloidal gold method), lead to insufficient sensitivity, and can only be applied to HIV positive patients, which greatly limits the promotion and application of this technology (Broger et al., 2020).

In this study, we aim to investigate the accuracy of LAM detection using a chemiluminescence assay. Therefore, we conducted LAM testing in patients diagnosed with TB (contain PTB and EPTB), non-TB, NTM and healthy control. The results showed that the positive rates in the four groups were 50.5%, 4.35%, 36.36%, and 5.48%, respectively (**Figure 2**). Moreover, the specificity of LAM in patients with Non-TB was 95.65%. Compared to previous studies, where LAM sensitivity in HIV-negative individuals ranged from 10% to 20% (van Crevel and Critchley, 2021; Liu et al., 2022), our study demonstrated significantly improved sensitivity. Our study also found that LAM had a sensitivity of 36.36% to NTM infection. These findings suggest that urine LAM testing for NTM infection may serve as a valuable adjunctive diagnostic tool but cannot differentiate between tuberculosis and NTM infections (De et al., 2020; Liu et al., 2022).

To determine potential factors affecting LAM detection results, we conducted univariate and multivariate logistic regression analysis on these risk factors, which have significant differences between LAM+ and LAM- groups. We found that diabetes and CD4 count were independent factors that influenced the results of LAM. Diabetes, a chronic metabolic disorder, the prevalence of PTB in the diabetic population is 2-3 times higher than in the normal population (van Crevel and Critchley, 2021), and diabetes mellitus may increase the risk of TB, with a 3.11-fold increase in the risk of PTB and a 1.18-fold increase in latent tuberculosis infection (LTBI) (Al-Rifai et al., 2017). Studies have reported that LAM enters the urine in the form of immune complexes (Sakamuri et al., 2013), with higher concentrations observed when renal filtration function is impaired (Lawn and Gupta-Wright, 2016). This phenomenon may explain why LAM is more easily detectable in individuals with tuberculosis and concomitant diabetes, as prolonged exposure to elevated blood glucose levels in diabetic patients can impair glomerular and tubular function (Oshima et al., 2021). Additionally, it has been suggested that impaired immune function in diabetic individuals may render them less capable of resisting the invasion and dissemination of Mtb (Birhanu et al., 2019). Since LAM concentration is closely related to bacterial load (Shah et al., 2010), this could contribute to the increased detectability of LAM in tuberculosis patients with diabetes. Furthermore, the association between a higher rate of LAM detection and a lower CD4 count has been extensively demonstrated in studies involving HIV-infected individuals (Huerga et al., 2019a). This may be attributed to the diminished anti-tuberculosis immune response associated with a low CD4 count, which leads to increased invasion and dissemination of Mtb, resulting in elevated LAM levels in the body (Huerga et al., 2019a). The WHO recommends the use of the Lateral Flow urine lipoarabinomannan (LF-LAM) assay as a rapid point-of-care test to assist in the diagnosis of tuberculosis in patients with advanced HIV-induced immunosuppression (Organization, 2019). Additional research also demonstrated that LAM exhibits higher sensitivity in patients with lower CD4 counts (Achkar et al., 2011) (Huerga et al., 2019b). Our study demonstrates that, when sensitivity is sufficient, LAM testing can also be applicable to HIV-negative populations and immunocompromised individuals.

We also compared LAM with other laboratory diagnostic methods. Our results showed that GeneXpert had a sensitivity of 55.13% and a specificity of 100%, The sensitivity of the two methods is in line with literature reports 57.4% (Zhang et al., 2018). TB-DNA had a sensitivity of 38.16% and a specificity of 100%; sputum culture had a sensitivity of 46.71% and a specificity of 100%. The sensitivity of the two methods is in line with literature reports (Horne et al., 2010; Inoue et al., 2011). Notably, the sensitivity of the urinary LAM assay (chemiluminescence) was determined to be 50.6%, which is significantly higher than that of TB-DNA(P<0.05), and higher than that of the sputum culture but lower than that of the GeneXpert method. These results suggest that the urinary LAM test may be a valuable tool for the diagnosis of tuberculosis, particularly in cases where GeneXpert or TB-DNA may not be readily available or suitable.

In addition, we also investigated the detection rates of the four diagnostic methods for pulmonary and extra pulmonary tuberculosis. Previous research has indicated that the incidence and prevalence of extrapulmonary tuberculosis may be underestimated due to several factors, including inadequate attention to extrapulmonary tuberculosis, insufficient diagnostic methods, and low rates of confirmation. This situation has led to a consistently lower cure rate for EPTB compared to PTB, posing challenges to global tuberculosis control efforts (Gambhir et al., 2017; Raghuvanshi et al., 2018). In this study, the detection rates of Gene Xpert, TB-DNA, and sputum culture for extrapulmonary tuberculosis are as follows: 11.76%, 16.67%, and 25%. Consistent with our findings, other studies have also observed that conventional methods are not very effective in detecting extrapulmonary tuberculosis, with a positivity rate ranging from 17.3% to 33.2% (Liu et al., 2020). The low sensitivity of these diagnostic tests may be attributed to the low number of mycobacteria present in peritoneal effusion (Rufai et al., 2015). In contrast, LAM showed detection rates of 51.7% for pulmonary tuberculosis and 42.11% for extrapulmonary tuberculosis. LAM’s detection rate in extrapulmonary tuberculosis was higher than the other three diagnostic methods, particularly significantly higher than Gene Xpert (P<0.05). This highlights the significant advantage of LAM in diagnosing EPTB compared to existing clinical diagnostic methods. It is non-invasive, simple, rapid, and accurate.

Our study has limitations that indicate further research is warranted. Firstly, it is a single-center study and did not cover data from multiple centers or a large sample size. Therefore, further research is needed to expand to broader regions and include a larger number of samples. Second, as a retrospective study, patients were not consequently recruited to complete the evaluation of multiple diagnostics. The bias in the selection of the participants may weaken confidence in results. Lastly, LAM testing cannot distinguish between TB and NTM, which is consistent with previous findings (Broger et al., 2020; van Crevel and Critchley, 2021). Overall, despite these limitations, our study highlights the potential for further investigation. Future research should encompass multiple centers, larger sample sizes, and prospective designs to ensure more comprehensive and robust results.

## Conclusion

In conclusion, urinary LAM antigen detection using chemiluminescence represents a significant advancement in the clinical diagnosis of active TB. It can be widely used in both HIV-negative and HIV-positive populations of tuberculosis patients. The superior diagnostic performance of LAM, combined with its convenience in acquiring samples from individuals who cannot produce sputum or have difficulties in sputum collection, makes it a promising approach. That can contribute to global efforts in controlling and managing tuberculosis. However, further research and validation are necessary to fully harness the potential of LAM detection and to optimize its integration into tuberculosis (TB) diagnostic algorithms worldwide.

## Data availability statement

The original contributions presented in the study are included in the article/**Supplementary Material**. Further inquiries can be directed to the corresponding author.

## Ethics statement

The studies involving humans were approved by Zhangjiagang Hospital (No. ZJGYYLL -2022-11-021). The studies were conducted in accordance with the local legislation and institutional requirements. Written informed consent for participation in this study was provided by the participants’ legal guardians/next of kin.

## Author contributions

LH: Writing – original draft. YN: Writing – review & editing. LZ: Writing – original draft. RY: Writing – original draft. MW: Writing – review & editing.

## Supplementary material

Supplementary Table 1 The diagnostic performance of four methods in NTM CRS, composite reference standard; NTM, nontuberculosis mycobacteria; Non-TB, non-tuberculosis; LAM, Liboarabinomannan;TB-DNA, *Mycobacterium tuberculosis* gene amplification assay; PPV, positive predictive value; NPV, negative predictive value. Sensitivity = True Positive Cases/(True Positive Cases + False Negative Cases) × 100%; Specificity = True Negative Cases/(True Negative Cases + False Positive Cases) × 100%; PPV = True Positive Cases/(True Positive Cases + False Positive Cases) × 100%; NPV = True Negative Cases/(True Negative Cases + False Negative Cases) × 100%.
