# Supplementary material for: Diagnostic value of chemiluminescence for urinary lipoarabinomannan antigen assay in active tuberculosis: insights from a retrospective study
Source: Front Cell Infect Microbiol. 2023 Dec 8;13:1291974. doi: 10.3389/fcimb.2023.1291974 (PMC10748405; doi:10.3389/fcimb.2023.1291974)
Supplement: Supplementary Table 1 — The diagnostic performance of four methods in NTM CRS, composite reference standard; NTM, nontuberculosis mycobacteria; Non-TB, non-tuberculosis; LAM, Liboarabinomannan;TB-DNA, Mycobacterium tuberculosis gene amplification assay; PPV, positive predictive value; NPV, negative predictive value. Sensitivity = True Positive Cases/(True Positive Cases + False Negative Cases) × 100%; Specificity = True Negative Cases/(True Negative Cases + False Positive Cases) × 100%; PPV = True Positive Cases/(True Positive Cases + False Positive Cases) × 100%; NPV = True Negative Cases/(True Negative Cases + False Negative Cases) × 100%. [file Table_1.pdf]

Supplementary Table 1. The diagnostic performance of four methods in NTM

Methods	Results	CRS		Sensitivity	Specificity	PPV	NPV	Kappa
		NTM	Non-TB					
LAM	positive	8	3	36.36%	95.65%	72.73%	82.50%	0.386
	negative	14	66					
Gene Xpert	positive	1	0	4.54%	100%	100%	76.14%	0.067
	negative	21	67					
TB-DNA	positive	6	0	27.27%	100%	100%	80.72%	0.361
	negative	16	67					
Sputum culture	positive	13	0	72.22%	100%	100%	93.15%	0.804
	negative	5	68					

CRS, composite reference standard; NTM, nontuberculosis mycobacteria; Non-TB, non-tuberculosis; LAM, Liboarabinomannan;TB-DNA, *Mycobacterium tuberculosis* gene amplification assay; PPV, positive predictive value; NPV, negative predictive value. Sensitivity = True Positive Cases / (True Positive Cases + False Negative Cases) × 100%;

Specificity = True Negative Cases / (True Negative Cases + False Positive Cases) × 100%; PPV = True Positive Cases / (True Positive Cases + False Positive Cases) × 100%;

NPV = True Negative Cases / (True Negative Cases + False Negative Cases) × 100%.
